# Engineered *Zymomonas mobilis* for salt tolerance using EZ-Tn5-based transposon insertion mutagenesis system

**DOI:** 10.1186/s12934-016-0503-x

**Published:** 2016-06-10

**Authors:** Jing-Li Wang, Bo Wu, Han Qin, Yang You, Song Liu, Zong-Xia Shui, Fu-Rong Tan, Yan-Wei Wang, Qi-Li Zhu, Yan-Bin Li, Zhi-Yong Ruan, Ke-Dong Ma, Li-Chun Dai, Guo-Quan Hu, Ming-Xiong He

**Affiliations:** Key Laboratory of Development and Application of Rural Renewable Energy (Ministry of Agriculture), Biomass Energy Technology Research Centre, Biogas Institute of Ministry of Agriculture, Section 4-13, Renmin Nanlu, Chengdu, 610041 People’s Republic of China; Xinjiang Production and Construction Corps Key Laboratory of Protection and Utilization of Biological Resources, College of Life Sciences, Tarim University, Tarim Basin, Alaer City, 843300 People’s Republic of China; Key Laboratory of Microbial Resources (Ministry of Agriculture, China), Institute of Agricultural Resources and Regional Planning, CAAS, Beijing, 100081 People’s Republic of China; College of Environmental and Chemical Engineering, Dalian University, Dalian, 116622 People’s Republic of China

**Keywords:** *Zymomonas mobilis*, Salt stress, Tn5-based transposon mutagenesis, Genome walking, *himA*

## Abstract

**Background:**

The cell growth and ethanol yield of *Zymomonas mobilis* may be detrimentally affected by salt stress frequently present in some biomass-based fermentation systems, leading to a decrease in the rate of sugar conversion to ethanol or other bioproducts. To address this problem, improving the salt tolerance of *Z. mobilis* is a desirable way. However, limited progress has been made in development of *Z. mobilis* with higher salt tolerance for some technical challenges in the past decades. Recently, transposon insertion mutant system has been widely used as a novel genetic tool in many organisms to develop mutant strains. In this study, Tn5-based transposon insertion mutagenesis system firstly used for construction of higher salt tolerance strain in *Z. mobilis*.

**Results:**

Approximately 200 *Z. mobilis* ZM4 mutants were generated by using Tn5-based transposon mutagenesis system. The mutant strain ZMT2 with improved salt tolerance phenotype was obtained by screening on RM agar plates with additional 1 % NaCl. Strain ZMT2 was confirmed to exhibit better fermentation performance under NaCl stress than wild type of strain ZM4. The transposon insertion was located in ZMO1122 (*himA*) by genome walking. Discruption of *himA* gene showed that *himA* may play an important role in response to salt tolerance in *Z. mobils*.

**Conclusions:**

The mutant strain ZMT2 with a transposon insertion in *himA* gene of the genome showed obviously higher sugar conversion rate to ethonal under up to 2 % NaCl stress than did the wild ZM4 strain. Besides, ZMT2 exhibited shared fermentative capabilities with wild ZM4 strain under no or low NaCl stress. This report firstly showed that *himA* played a role in responding to NaCl stress. Furthermore, the result indicated that Tn5-based transposon mutagenesis system was a feasible tool not only for genetic engineering in *Z. mobilis* strain improvement, but also in tapping resistent genes.

## Background

*Zymomonas mobilis* is a desirable ethanologenic bacterium with many excellent industrial characteristics [1–4]. Although *Z. mobilis* exhibited valuable properties, such as higher ability to convert sugar to ethanol compared to its low biomass [5], the strain was sensitive to inorganic salt inhibitors added to or produced from fermentable lignocellulosic hydrolysates and biodiesel wastes [6–11]. Previous studies indicated that NaCl is a common inhibitor in *Z. mobilis* during fermentation [9]. On the one hand, cell morphology of *Z. mobilis* has been reported to form adverse filaments under salt stress condition. On the other hand, cell growth and fermentation are also detrimentally affected by salt stress, resulting in reduced rate of sugar conversion to ethanol. This deficiency made it failure to meet industrial needs. If fermented substrates are subjected to desalination process before fermentation, input will increase proportionally. Therefore, engineering a higher salt tolerance *Z. mobilis* strain is a preferable way.

Concerns on development of stress tolerance *Z. mobilis* strain have been growing in the past decades. Previously published reports mainly focused on two strategies, namely adaptive laboratory evolution (ALE) and rational engineering strategies. ALE has been successfuly applied in *Escherichia coli* [12], *Saccharomyces cerevisiae* [13], as well as *Z. mobilis* [14–16] to obtain prospective phenotypes. But in our previous attempt to develop higher salt resistance in *Z. mobilis* by ALE, the culture could not reproduce for continuous passage under 1.5 % NaCl stress. The process was rather blind and long-lasting in that ALE does not modify a certain phenotype in a fashion of trimming related genetic mechanism. The trial failed probably because some key factors were absent in the medium acting as precursors of metabolites counterbalancing osmotic pressure. Up to date, the mechanisms of stress response are still not unveiled, which are basic requirements for rational engineering strategies. Under this circumstance, engineered salt tolerance *Z. mobilis* strain calls for new methods.

Recently, transposons have been developed to be prevalent molecular tools for both whole-genome and single-gene studies in bacteria, yeast, and other microorganisms [17–20]. A few meaningful attempts have been made, which introduced transposons with target heterogenous genes into *Z. mobilis* genome to get desirable phenotypes, i.e, xylose utilization, heat resistance and growth under malnutrition [21, 22]. However, native genes with transposon insertion may also affect the phenotype, which was ignored in the two papers above. Actually, the genome annotation of *Z. mobilis* ZM4 showed that open reading frames (ORFs) coding genes with assigned putative functions only account for 67.4 % of total coding ORFs, leaving an important percentage unknown [3]. Functional unknown genes may be candidates for salt tolerance.

So, in this study, we focused on engineering a recombinant strain with improved salt tolerance phenotype via EZ-Tn5-based transposon insertion mutagenesis system. Then, the mechanism of enhanced salt resistance in the engineered strain may lay foundations for the study of stress resistance in *Z. mobilis*. Furthermore, this research could give a proof that this mutagenesis system was a feasible tool for *Z. mobilis* improvement.

## Results and discussion

### Screening of improved NaCl tolerance mutants from *Z. mobilis* ZM4 mutant library

After 3 days growth, *Z. mobilis* ZM4 mutant library for about two hundred transformants were obtained with chloramphenicol resistance. The library was gathered by washing the plates using liquid RM medium and stored in −80 °C. For further screening, partial cultures were plated on RM agar medium with 1 % (w/v) NaCl after overnight recovery. Two clones were found on the stress plate and designated as ZMT1 and ZMT2 (stored at China General Microbiological Culture Collection Center, CGMCC No. 11888). Our previous study showed that wild type ZM4 could not grow on supplemented with 1 % (w/v) NaCl RM agar medium (data not shown). It indicated that ZMT1 and ZMT2 have higher salt tolerance than wild type of ZM4. Further verification was also carried out by normal PCR with the primer pair of cm-1-5′ and cm-1-3′. Expectly, about 900 bp Cm fragments were amplified with primer pair of cm-1-5′ and cm-1-3′ from the chromosomal DNA of ZMT1 and ZMT2, respectively. However, no fragment amplified with the same primer pair from the chromosomal DNA of ZM4. These results indicated that the transposon should have integrated into the genomes of ZM4, and produced two mutants (ZMT1 and ZMT2).

### Location of transposon insertion by genome walking

**Fig. 1:**
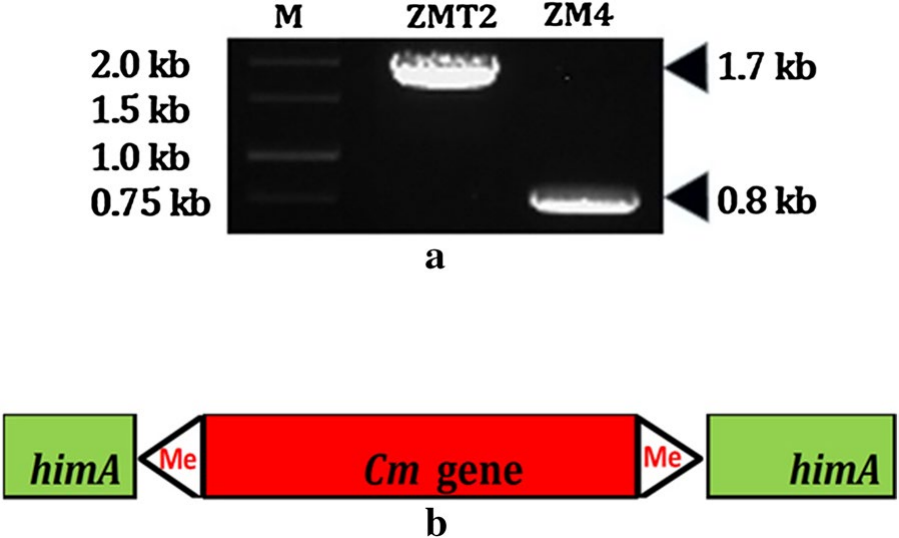
PCR analysis of transposon insertion in ZMT2 and ZM4 as control. **a** The size of the PCR product corresponding to the fragment between ZMO1122 forward and ZMO1121 downward is indicated on the *right*. *M* indicates the 1 kb DNA ladder. **b** Structural diagram of transposon in ZMT2 genome. *Me* mosaic end

For verification of its location of transposon insertion in ZM4 genome, genome walking technology was also performed according to procedure of the kit. ZMT2 was chosen for further studies for its better growth than that of ZMT1 when exposure to NaCl stress. The stability of integration in ZMT2 was also tested by several passages in RM liquid without stress. After subsequently three nested PCR, a prominent electrophoresis band was isolated, extracted and sequenced. For the downstream DNA fragment adjacent to *cm* gene, the alignment showed that the sequence shared 100 % similarity with 5′ region of ZMO1122 (*himA*) in ZM4 genome by BlastN program. The alignment of upstream DNA fragment had an agreement with the conclusion that the insertion was located in *himA* in ZMT2 genome. The confirmation was carried out by PCR with primers of 1122-5′ and 1121-3′ and gDNA of ZMT2 and ZM4 as templates, resulting in about 1700 and 800 bp, respectively (Fig. 1a). The larger fragment contained *Cm* gene (as shown in Fig. 1b), which was verified by sequencing. The result also indicated that the mutant was very stable by transposon insertion into the genome, and the same speculation was also reported by Jia [22]. Taken together, transposon insertion mutagenesis system may be a useful tool for construction of a stable strain.

### Disruption of the *himA* gene in *Z. mobilis* ZM4

**Fig. 2:**
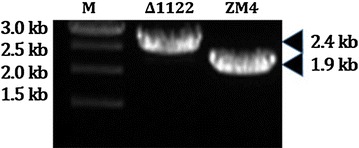
PCR analysis of disruptant *himA* in Δ1122 and ZM4 as control. The size of the PCR product is indicated on the *right*. *M* indicates the 1 kb DNA ladder

With the transposon insertion, the function of wild gene was seriously affected, because the gene was fracture in two parts and between was *cm* cassette. However, the relationship between *himA* and NaCl tolerance was not reported in previously published papers. Whether *himA* gene played a role in response to NaCl stress or not needed futher supports. For understanding of *himA* gene function, gene disruption is necessary. For the disruption of the *himA* gene, reconstruction plasmid pJect-Δ1122 habouring the 5′- and 3′-regions of *himA* gene linked with *cm* cassette, and then transformed into *Z. mobilis* ZM4. Based on *cm*-resistance phenotype, one isolate designated as *Z. mobilis* Δ1122 was chosen for disruption analysis of the *himA* gene. The result was firstly confirmed by the marker gene using primers cm-1-5′ and cm-1-3′. The confirmation was also performed to ensure the disruption of the *himA* in *Z. mobilis* Δ1122 by PCR using specific primers 1122-up-5′ and 1121-down-3′. The PCR product of approximately 2.4 kb was observed in the *Z. mobilis* Δ1122, and in *Z. mobilis* parental strain was 2.0 kb, which was sequenced and identical to the expectation (Fig. 2).

### Analysis of cell growth, glucose utilization and ethanol product

For cell growth analysis, the mutant strain ZMT2 with a transposon insertion in *himA* gene showed obviously higher sugar conversion rate to ethonal under up to 2 % NaCl stress than did the wild strain ZM4. With 1.5 % NaCl stress, the glucose utilization rate in ZMT2 was obviously faster than that in ZM4, but ethanol yield rate was not increasing proportionally in ZMT2. When cultured in less than 1.5 % NaCl, ZMT2 and ZM4 exhibited almost shared features in cell growth, glucose utilization and ethanol product.

During cell growth analysis, a strange finding was that salt stress resistance of all the three strains decreased when they were cultured on RM agar medium compared to in liquid medium. For example, both ZMT2 and Δ1122 could grow on the plate with 1 % NaCl, but ZM4 not. A reasonable assumption was that NaCl stress was related to cellular dissolved oxygen in facultative anaerobic *Z. mobilis*. Cellular dissolved oxygen was supposed to affect respiratory chain in that it was reported to provide salt stress tolerance by maintaining a low NADH/NAD^+^ ratio in *Z. mobilis* [23]. Based on fermentational assessments, the mutant strain ZMT2 with a transposon insertion in *himA* gene showed obviously higher sugar conversion rate to ethonal under up to 2 % NaCl stress than did the wild strain ZM4. After determination, the mutant was very stable without antibiotic stress, which can be more economical in industrial fermentation. Therefore, strain ZMT2 may act as a host for further engineering modification to develop multiple desirable phenotypes, such as resistance to various stress, capacity to simultaneously convert glucose and xylose and synthesis of byproduct with high additional value.

**Fig. 3:**
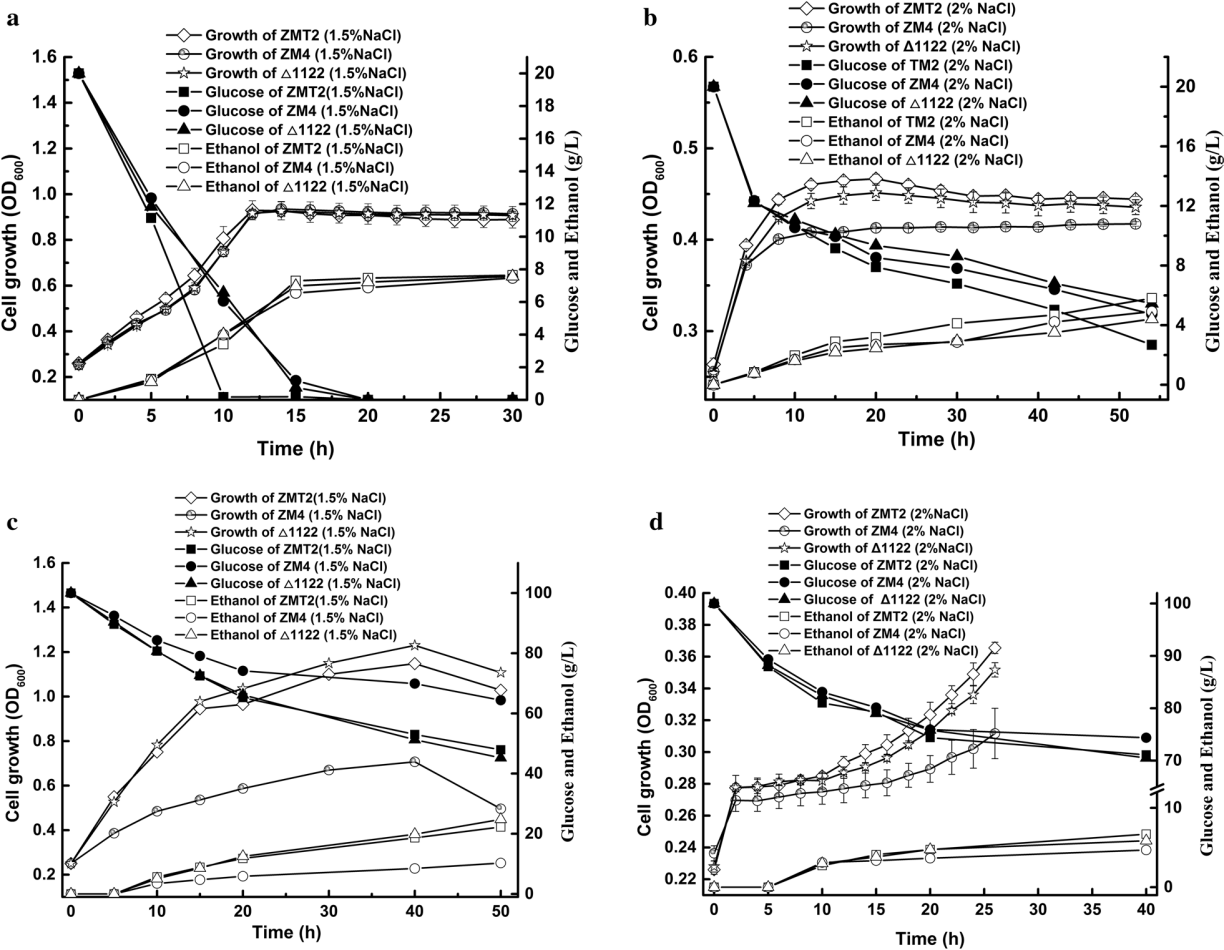
Cell growth, glucose utilization and ethanol production in ZMT2, ZM4 and Δ1122 media with different NaCl stress. **a** The medium contains 20 g/L glucose and 15 g/L NaCl. **b** The medium contains 20 g/L glucose and 20 g/L NaCl. **c** The medium contains 100 g/L glucose and 15 g/L NaCl. **d** The medium contains 100 g/L glucose and 20 g/L NaCl

The quantification of glucose utilization and ethanol product with HPLC showed that significant differences of glucose utilization rate and ethanol product lied in the NaCl stress up to 1.5 and 2.0 %, and that ZMT2 possess best fermentation properties under higer NaCl stress compared to ZM4 and Δ1122 (Fig. 3). When fermented in RM with 1.5 % NaCl, ZMT2 almost used up the glucose in the medium within 10 h, but ZM4 and Δ1122 just used 69.70 and 67.15 % of the total glucose under the same condition at 10 h. The ethanol product reached 7.29, 6.54 and 6.97 (g/L) with ZMT2, ZM4 and Δ1122 by 15 h culture. Until by 20-h culture, the conversion of total glucose by ZM4 and Δ1122 reached 100 %. And the maximum product were 7.77, 7.57 and 7.62 (g/L), respectively in ZMT2, ZM4 and Δ1122 fermentation broth by 66 h. Under 2 % NaCl stress, the remaining glucose concentrations were 5.30, 16.75 and 22.50 %, respectively in ZMT2, ZM4 and Δ1122 culture broth by 66 h, resulting in ethanol products of 6.83, 5.46 and 5.14 (g/L). Researches on cell growth, glucose utilization and ethanol product of the thress strains were also conducted in 10 % glucose RM with serial NaCl stress. Specially, mutants showed obvious advantages on glucose utilization and ethanol product over ZM4 under 1.5 % NaCl stress. Detailed results were shown in Fig. 3.

From the comprehensive results above, strain ZMT2 was superior to ZM4 and Δ1122 in coping with NaCl stress. Meanwhile, the data indicated that glucose utilization was not coupled with ethanol product as previously reported [24]. For ZMT2 under 1.5 % NaCl, the increasing utilization of glucose was not likely in proportion to its biomass and ethanol product. An assumption was that a part of glucose was converted to synthesis of something like compatible solutes, which were reported to play a key role in response to NaCl stress in most halophiles [25–27]. We conceived that *himA* gene was supposed to contribute to a certain pathway in ZMT2 to balance the osmotic stress. Since 2 % NaCl posed a serious threat to the normal growth, regulating capacity responding to NaCl stress became too poor to maintain normal growth for all the three strains.

### Real-Time qPCR analysis of *himA*, *pdc* and *adhB* genes under NaCl stress

*HimA* gene expression was not detected in ZMT2 and Δ1122 with or without NaCl stress by Real-Time qPCR, which indicated the disruption of *himA* in ZMT2 and Δ1122. However, noticeable differential expression was found in ZM4. The normalized fold expression of *himA* under 1.5 % NaCl in ZM4 was 3.18 ± 0.88, which was nearly six times as those under NaCl concentrations of 0, 1 and 2 % with expressions of 0.56 ± 0.17, 0.55 ± 0.39 and 0.54 ± 0.28, respectively (Table 1). We deduced that 2 % NaCl stress damaged cell growth to a large extent and as well as *himA* gene expression.

**Table 1:** Comparison of different gene expression by qPCR

Gene	Strain	NaCl concerntration (w/v) (%)	Expression
*adhB*	TM2	0	0.014 ± 0.039
		1	0.044 ± 0.098
		1.5	0.417 ± 0.262
		2	8.126 ± 2.413
	ZM4	0	0.073 ± 0.060
		1	0.070 ± 0.103
		1.5	0.235 ± 0.235
		2	1.000 ± 0.753
	Δ1122	0	1.533 ± 0.654
		1	2.054 ± 1.838
		1.5	1 ± 1.067
		2	32.026 ± 23.792
*pdc*	TM2	0	1.223 ± 0.322
		1	0
		1.5	0.260 ± 0.099
		2	1.832 ± 1.091
	ZM4	0	0.646 ± 0.199
		1	0.538 ± 0.105
		1.5	0.311 ± 0.177
		2	0.326 ± 0.257
	Δ1122	0	1.533 ± 0.677
		1	0.214 ± 0.047
		1.5	0.112 ± 0.067
		2	3.466 ± 4.705
*himA*	ZM4	0	0.558 ± 0.165
		1	0.553 ± 0.394
		1.5	3.179 ± 0.876
		2	0.543 ± 0.281

Actually, integration host factor subunit alpha (HIMA) encoded by ZMO1122, acted as DNA repair and recombination proteins. Previous reports showed that *himA* gene was found to be involved in the inhibitory effect of acetate on *Zymomonas* performance [28, 29], but the relationship between *himA* and salt tolerance was not reported. Under salt stress, the process of DNA recombination may be much easier to go wrong, which needed more repair proteins like HIMA to ensure the fidelity of DNA synthesis. Therefore, *himA* gene expression in ZM4 was higher with 1.5 % NaCl stress than 0, 1 and 2 % NaCl. In 2 % NaCl, the cell growth was badly inhibited leading to various metabolisms retarded, resulting in quite low expression of *himA* gene. But the acquistision of improved salt tolerance in ZMT2 was probablely via another pathway. Disruption of *himA* may facilate balancing the osmotic pressure by producing or accumulating counterpoise metabolites. Given that the detailed mechanism was well-understood, the illumination of stress tolerance would be more evident.

*Pdc* gene expression was also found apparent changes with NaCl stress. In normal RM medium, normalized fold expressions of *pdc* gene in ZMT2 and Δ1122 were 1.893 and 2.373 times as that in ZM4 (0.646 ± 0.199), respectively. Under 1 and 1.5 % NaCl concentration, *pdc* gene expressions decreased sharply in the three strains. However, 2 % NaCl seemed to greatly stimulate *pdc* expression in ZMT2 and Δ1122, which was 4.62 and 9.63 times higher than that in ZM4 (0.33 ± 0.26).

With regard to *adhB* gene expression, the trend was that the expression was increased with the increasing of NaCl concentration. Compared with normalized culture, the expressions in the three strains were obviously up-regulated under 2 % NaCl stress. Specifically, *adhB* gene expressions under 2 % NaCl in ZMT2 and Δ1122 were 8.13 and 32.03 times as that in ZM4. In general, the normalized fold expression was the highest in strain Δ1122 with or without NaCl stress among the three strains.

### Enzyme activity assays of ADHB and PDC under NaCl stress

To verify the *adhB* and *pdc* gene expression, related enzyme activities were also detected. In line with quantitative detection result of *adhB* gene, ADHB activities showed similar tendency with the NaCl stress in the three strains (Table 2), which were higher under 1.5 % NaCl than 0, 1 and 2 % NaCl. The maximum ADHB activities were basically identical among ZMT2, ZM4 and Δ1122 which were respective 38.0, 41.25 and 98.56 times as those without NaCl stress. Under 2 % NaCl stress, the ADHB in wild type strain ZM4 showed the lowest activity with 15.57 U/g, and in ZMT2 and Δ1122 with 47.69 and 42.62 U/g. ADHB activities were still higher under 2 % NaCl than without NaCl which were suitable for the three strains. A balance was between ADHB activity and NaCl stress, only certain NaCl stress inducing higher activity of ADHB.

**Table 2:** Comparison of ADHB and PDC activity in different strains under NaCl stress

Strains	NaCl (w/v) (%)	ADHB activity	PDC activity
ZMT2	0	14.099 ± 9.600	41.776 ± 0
	1	278.399 ± 26.482	17.070 ± 0.732
	1.50	535.834 ± 46.239	14.969 ± 3.583
	2	47.687 ± 7.330	9.676 ± 1.955
ZM4	0	13.055 ± 9.600	22.978 ± 2.954
	1	314.184 ± 42.534	38.159 ± 0.981
	1.50	546.630 ± 22.154	3.309 ± 0
	2	34.907 ± 15.574	6.234 ± 2.939
Δ1122	0	5.724 ± 0.736	31.221 ± 2.943
	1	269.784 ± 44.713	40.770 ± 1.177
	1.50	564.142 ± 69.869	21.153 ± 2.601
	2	42.617 ± 11.995	10.390 ± 1.690

However, that was not the case for PDC activity. The increased NaCl stress leaded to reduced PDC activity in the strains. In ZMT2, PDC activity was linearly reduced as NaCl increasing. In ZM4 and Δ1122, the activities were higher under 1 % NaCl than 0, 1.5 and 2 % NaCl. With 2 % NaCl, the activity in Δ1122 was the highest (10.39 U/g), followed by ZMT2 (9.68 U/g) and ZM4 (6.23 U/g).

The data demonstrated that enzyme activities of ADHB and PDC were not in proportion of the expression of corresponding genes, because enzyme kinetic parameters and stability were partially affected by in vivo salt concentration. The enzyme activity assays were generally in agreement with the RT-qPCR of *adh*B and *pdc* genes under different NaCl stress. With salt stress, PDC and ADH activities the key enzymes in Entner-Doudoroff (ED) pathway were affected with different degrees, which represented changes in carbon center flux.

### Quantification of NADH/NAD^+^

Without salt stress, intracellular reduced nicotinamide adenine dinucleotide (NADH) was the overwhelmingly dominant form in the three strains. And NADH/NAD^+^ ratios in ZMT2 and Δ1122 were respectively 4.93 and 11.74 times higher than that in ZM4 (as shown in Table 3). Once cultured under 1.0 % NaCl stress, the ratios decreased dramatically as low as 0.28, 0.01 and 0.18 in ZMT2, ZM4 and Δ1122 respectively. With 1.5 % NaCl stress, intracellular oxidized nicotinamide adenine dinucleotide (NAD) was the absolutely predominant form for all strains. Up to 2.0 % NaCl, however, the ratios had a slightly increasing.

**Table 3:** NADH/NAD^+^ ratio under different NaCl stress

NaCl (w/v) (%)	Strain	NADH/NAD^+^
0	ZMT2	382.49
	ZM4	77.58
	Δ1122	910.53
1.0	ZMT2	0.28
	ZM4	0.01
	Δ1122	0.18
1.5	ZMT2	0
	ZM4	0
	Δ1122	0
2.0	ZMT2	0.19
	ZM4	0.13
	Δ1122	0.08

Previously published reports showed that NADH and NAD were involved in numerous life activities, such as regulation of corepressor function by nuclear NADH [30], NAD-dependent enzyme related to DNA repair [31], NAD-utilizing metabolic pathways potentially acting in RNA repair [32] and *Z. mobilis* lost NADH dehydrogenase activity producing high levels of ethanol [33]. Especially in *Z. mobilis*, Takeshi Hayashi [23] reported a low NADH/NAD^+^ ratio providing salt stress tolerance, which was in agreement with our results. Regarding the ratios, an obvious trend was that the values were the lowest in ZM4 with or without NaCl strss, compared to the other two strains. We reasoned that higher NADH/NAD^+^ ratio facilitated to counteract the disruption of *himA* in ZMT2 and Δ1122 without salt stress. On the other hind, when cells were exposed to salinity, low NADH/NAD^+^ ratio as one promoting factor contributed to salt tolerance, which could maintain a comparative higher level with the help of the disruption of *himA* as another factor to balance the stress in mutants. Therefore, ZM4 cells had the ratio lower enough to keep balancing than that in ZMT2 and Δ1122. Further researches were needed to uncover the potential mechanism of NADH/NAD^+^ responding to salt stress.

## Conclusions

An engineered *Z. mobilis* ZMT2 strain with increased salt tolerance was obtained by Tn5-based transposon insertion mutagenesis system. In ZMT2, ZMO1122 (*himA*) gene was destroyed as the transposon inserted. Disruption of ZMO1122 gene in wild type strain ZM4 achieved similar phenotypes, which proved *himA* acting in responding to NaCl stress. The mechanism of salt tolerance in ZMT2 promoted to throw light on responses to unfavorable conditions in microorgism. Furthermore, the result indicated that Tn5-based transposon is a feasible tool for strain breeding in *Z. mobilis.*

## Methods

### Strains, plasmids and culture conditions

*Zymomonas mobilis* ZM4 (ATCC 31821) was used as the wild type strain and engineered salt tolerance in this study. Mutant ZM4 strain with EZ-Tn5-based transposon insertion was designated as *Z. mobilis* ZMT1 and ZMT2. Strain ZM4 Δ1122 was disrupted ZMO1122 (*himA)* gene. All the strains was cultured in Rich media (RM) [34] at 30 °C without shaking. When necessary, certain proportion of NaCl (1, 1.5, 2 %, w/v) was separately added to RM medium. Cultures were stored by suspending in 25 % (v/v) glycerol at −80 °C for long-term preservation. For the extraction of nucleic acids, strains were grown in RM medium at 30 °C. After 16 h of incubation, cells were harvested by centrifugation at 4000 rpm for 3 min and washed twice with sterilized distilled water. *E. coli* strain DH5α was used for gene manipulation. It was grown in Luria-Bertani (LB) medium at 37 °C for 14 h.

Transposon Construction Vectors EZ-Tn5 pMOD™-2 in the EZ-Tn5™ Custom Transposome Construction Kits (Epicentre^®^, WI, USA), was used to generate novel Tn5-based transposons and ZM4 mutant pool. Clone vector pZeroBack (pJect) (TIANGEN Biotech, Beijing, China) was used to clone DNA fragments and to disrupt the *Z. mobilis* ZMO1122 gene in fusion PCR-based construction experiments. This vector contains the ampicillin (Amp) resistant marker gene. The chloramphenicol (*cm*) resistant gene was cloned from pLysS vector (Novagen, Madison, WI, USA).

### Construction of Tn5 transposon and ZM4 genome mutant pool

The pLysS vector was transformed into electrocompetent *E. coli* DH5α by electroporation. The positive clone was selected based on a chloramphenicol resistance marker. Plasmid DNA was extracted by E.Z.N.A.^®^ Plasmid Mini KitI (Omega Bio-Tek, Norcross, GA, USA) and used as PCR template for isolation of *cm* gene. The primers with restrict enzyme sites, cm-1-5′ (*Pst*I) and cm-1-3′ (*Sac*I) were list in Table 4. The recombinant pJect-cm vector was obtained by Zero Back Fast Ligation Kit and was then subjected to sequencing by Genewiz Company (Suzhou, China). After *Pst*I and *Sac*I double digestion, *cm* gene was ligated into pMOD-2 vector digested by the same restriction enzymes, to generate pMOD-2-cm vector. The positive clone was selected on LB agar medium with chloramphenicol stress. The recombinant pMOD-2-cm plasmid from the positive clone was confirmed by sequencing and used as PCR template with the primer pair ME Plus 9-5′ and ME Plus 9-3′. The generation was linear DNA fragment containing hyperactive 19-bp Mosaic Ends (ME) and *cm* gene replaced of the multiple cloning site (MCS) sequence between *Pst*I and *Sac*I restriction sites. The DNA fragment was purified by E.Z.N.A.^®^ Gel Extraction Kit (Omega Bio-Tek, Norcross, GA, USA) and modified by Tn5 transposase to facilitate itself to integrate into ZM4 genome. The Tn5 transposome reaction was performed according to the product manual. The final transposon was then transformed into competent ZM4 by Gene Pulser Xcell ^TM^ Electroporation System (Bio-Rad, Hercules, CA, US). For preparation of the competent cells, fresh cells were processed as described by Zhang [21]. Electroporation was performed under the condition of 1.5 kV, 5.0 ms, in 0.1-mm gapped electroporation cuvettes with 50 μL competent cell and 1 μL transposon mixture. The transformants were screened on RM agar plates with 34 (μg/mL) chloramphenicol. The positive clones was gathered in liquid RM medium and further screened on RM agar medium with 1 % (w/v) NaCl, because our previous study showed that the growth of wild type ZM4 was seriously hindered under this condition.

**Table 4:** List of primers used in the study (restrict sites underlined)

Gene	Primer	Sequences (5′–3′)	Usage
ME Plus 9	ME Plus 9-5′	CTGTCTCTTATACACATCTCAACCCTGA	Insertion mutant
	ME Plus 9-3′	CTGTCTCTTATACACATCTCAACCATCA	
pLys *cm*	cm-1-5′	AACTGCAGTATCACTTATTCAGGCGTAGCA	
	cm-1-3′	CGAGCTCAATAAATCCTGGTGTCCCTGT	
	cm-SP1-5′	ATTCACATTCTTGCCCGCCTGATG	Insertion location
	cm-SP2-5′	AGACGGTGAGCTGGTGATATGGGAT	
	cm-SP3-5′	GAATACCACGACGATTTCCGGCAGT	
	cm-SP1-3′	CGAAGAAGTTGTCCATATTGGCCACG	
	cm-SP2-3′	AAACCCTTTAGGGAAATAGGCCAGGT	
	cm-SP3-3′	TCACTCCAGAGCGATGAAAACGTTTC	
	cm-2-5′	TATCACTTATTCAGGCGTAGCA	Gene disruption
	cm-2-3′	AATAAATCCTGGTGTCCCTGT	
ZMO1122	1122-5′	ATGAATAAACGCTATGATAACCGCAC	
	1122-3′	TTAGGCTCCCTTGATAATCCGC	
	1122-up-5′	CGTTCAGCAGCCATATTTCTAGCCT	
	1122-up-cm-3′	CAGGGACACCAGGATTTATTCGCAAGTAGGTGATTCTAAT	
	1122-down-cm-5′	GTGCTACGCCTGAATAAGTGATACTTGTCTGATCGCGATGCCC	
	1122-down-3′	AAGTCCCGCCCTTCTTTCCAAC	
	1122-F	TGAATAAACGCTATGATAAC	qPCR
	1122-R	ATCTGTAATGTGACCAAG	
*adhB*	adhB-F	TACAACCTCGTCCTTCTT	
	adhB-R	CATAACCTTCTGCACTGA	
*pdc*	pdc-F	GGTATTAATTCTGCTGTT	
	pdc-R	CGAAGTCTGAATTGTTAT	
16S rDNA	16S-F	TCAACTATAGACCAGTAAGT	
	16S-R	AGAACATAGAAGAGGTAAGT	

### Location of transposon insertion in ZMT2 gDNA by genome walking

The transformants ZMT2 with improved NaCl stress was chosen for further study. The genome DNA was isolated from strain ZMT2 and ZM4 using E.Z.N.A. ^TM^ Bacterial DNA Kit (Omega Bio-Tek, Norcross, GA, USA). The Tn5 insertion within the genome was verified by PCR with the primer pair cm-1-5′ and cm-1-3′, as well as ME Plus 9-5′ and ME Plus 9-3′.

The insertion location was detected in ZMT2 by Genome Walking Kit (TaKaRa, Otsu, Shiga, Japan) according to the instruction manuals [35]. The downstream DNA fragment adjacent to *cm* gene was amplified by subsequently three nested PCR with successively combined use of specific primers cm-SP1-5′, cm-SP2-5′ and cm-SP3-5′ and a certain annex primer (AP). The PCR product was diluted by 100 times when used as the template for next PCR reaction. Likewise, the upstream DNA fragment was obtained with specific primers cm-SP1-3′, cm-SP2-3′ and cm-SP3-3′ and an AP. Both of the third PCR product was purified and sequenced by Genewiz Company. The sequencing results were analyzed by BLASTN program in NCBI (National Center for Biotechnology Information) online database.

### Disruption of the *himA* gene in *Z. mobilis* ZM4

The gene disruption construction was carried out by the PCR-based procedure [36]. Specific primers were designed as shown in Table 4. Firstly, the 5′- and 3′-regions of *himA* gene, as well as *cm* cassette were amplified via standard PCR. Primer pairs for the 5′-*himA* and 3′-*himA* were 1122-up-5′, 1122-up-cm-3′, 1122-down-cm-5′ and 1122-down-3′, resulting in a 662 bp 5′-region and an 837 bp 3′-region. The primers of cm gene were cm-2-5′ and cm-2-3′ to generate an 889 bp fragment. Secondly, fusion PCR, after the three fragments purified, 5′-region, 3′-region and *cm* cassette were added into the reaction system by an equal volume. PCR product was sequenced, followed by transformation into competent ZM4 cells. Tranformants were screened on RM agar plates with 34 (μg/mL) chloramphenicol. After 3 days incubation at 30 °C, positive clone was identified by normal PCR and nested PCR, based on the marker gene sequence.

### Analysis of cell growth, glucose and ethanol under NaCl stress

Cell growths of ZMT2, ZM4 and Δ122 were monitored by BioScreen C instrument (Lab Systerms, Helsinki, Finland) in 2 and 10 % glucose RM medium with different NaCl concerntrtion (0, 1, 1.5, 2 %, w/v) with three replicates, respectively [10, 37, 38]. Otherwise additional illustration, cell growth was carried on in 2 % glucose RM medium. Revived cells were adjusted to be of the same OD_600_, 30 μL seed culture added to 270 μL medium with an original OD_600_ of about 0.25. The running condition was at 30 °C and medium shaking, in case of biomass precipitation at the bottom of the wells. The absorbance readings were periodically taken every 2 h for total 30 times. Cell growth was also determined by measuring the optical density at 600 nm by a UV765 spectrophotometer at 5-h intervals.

The determination of glucose utilization and ethanol product in ZMT2, ZM4 and Δ122 under different NaCl stress (0, 1, 1.5, 2 %, w/v) was performed by High-performance liquid chromatography (HPLC) (Agilent Hi-plex H, 300 mm × 7.7 mm) with sulfuric acid (0.05 M) as mobile phase at a flow rate of 0.6 mL/min and a column temperature of 35 °C. The quantification was dependent on the linear relationship between the stripping peak height and the concentration of glucose and ethanol, respectively, corresponding standards as references. 35 μL cultures were incubated in 50 mL centrifuge tubes with an original OD_600_ of 0.25, at 30 °C no shaking. During fermentation, 1 mL fermentation broths were taken every 5 h, centrifuged to withdraw culture supernatant and then stored at −20 °C after filtered by 0.2 μm membrane (Millipore).

### Real-time qPCR analysis of *himA*, *pdc* and *adhB* genes under NaCl stress

Because pyruvate decarboxylase (*pdc*) and alcohol dehydrogenase B (*ahdB*) were key emzymes for ethanol product, the expression of corresponding genes in ZMT2, ZM4 and Δ122 under different NaCl stress (0, 1, 1.5 %, w/v) was determined by real-time RT-PCR [39]. Strains were gathered when the growths approached OD_600_ = 1. For total RNA preparation, E.Z.N.A.™ Bacterial RNA Kit (Omega Bio-Tek, Norcross, GA, USA) was used. The quality and integraty of RNA was assessed by NanoVue™ Plus (GEHC, USA) and agarose gel electrophoresis.

First-strand cDNA synthesis and quantitative real-time reverse transcription PCR system were conduted by QuantScript RT Kit (with gDNase) and SuperReal PreMix Plus (SYBR Green) (TIANGEN, Beijing, China) following the manufacturer’s instruction. The RT-qPCR amplifications were performed in the Bio-Rad iQ™5 real-time PCR detection system (Bio-Rad, Hercules, CA, US). The reactions were carried out in a total volume of 20 μL containing 10 μL 2× SuperReal PreMix Plus, 1 μL diluted cDNA, 7.8 μL sterile water and 0.6 μL of each forward and reverse primer. The RT-qPCR running conditions were as follows: 95 °C for 15 min, followed by 40 cycles at 95 °C for 10 s, 55–60 °C for 20 s and 72 °C for 20 s, and final extension at 72 °C for 5 min. The expression of 16S rDNA was chosen as an internal control with specific primers as shown in Table 4. DNase-free H_2_O replaced cDNA template as a negative control. Target gene expression data was calculated with 16S rDNA as the reference gene by IQ5 Optical System Software (Version 2.1, Bio-Rad, USA) according to the Real-Time PCR Applications Guide. All experiments were in triplicate. All data provided are the mean of corresponding mRNA expression (Table 1).

### Enzyme activity assays of PDC and ADHB under NaCl stress

Strains ZMT2, ZM4 and Δ122 were independently seeded in RM medium supplymented with NaCl concerntration (0, 1, 1.5, 2 %, w/v). After incubated for 12 h, cells were harvested by centrifugation at 4000*g*, 4 °C for 2 min and subjected to lysis by Yeast Protein Extraction Kit (CWBIO, Beijing, China) following the product manual. The supernatant was isolated by centrifugation at 12,000 rpm, 4 °C for 10 min and stored at −20 °C for subsequent assays. Raw protein concerntrations were determined based on the linear relationship between optical density at 595 nm and protein concerntrations, with bovine serum albumin (BSA) as the Ref. [40].

PDC activity was indirectly determined by monitoring the decrease of OD_340_ in a period, which reprented the pyruvic-acid dependent oxidation rate of NADH [41]. The reaction system was carried out at 25 °C, which consisted of (μL) 200 mM sodium citrate buffer (pH 6.0) 925, 10 mg/ml NADH (sodium salt, Sigma, USA) 10, 100 mg/ml sodium pyruvate 32, 10 mg/ml alcohol dehydrogenase (Solarbio, Beijing, China) 3, enzyme sample 30. One unit of enzyme activity is defined as the conversion of 1.0 μM pyruvate to acetaldehyde/min at pH 6.0 and 25 °C.

Likewisely, the determination of ADHB activity was indirectly computed by monitoring the increasing rate of OD_340_, corresponding to the oxidation rate of ethonal into ethylal [41]. The reaction system contained (μL): 35 mM Trizma base (pH 8.5) 930, 20 mg/ml NAD^+^ 30, absolute ethanol 30, enzyme sample 10, and was conducted at 25 °C. One unit of enzyme activity is defined as the conversion of 1.0 μM ethanol to acetaldehyde/min at pH 8.5 and 25 °C.

### Quantification of NADH/NAD^+^

About 2 × 10^5^ cells of ZMT2, ZM4 and Δ1122 under different NaCl stress (0, 1, 1.5, 2 %, w/v) were pelleted when cultured to middle-logarithmic phase [23]. Cells were washed with cold PBS, followed by resuspending in 400 μL NADH/NAD Extraction Buffer from NAD/NADH Quantification kit (Sigma-Aldrich, St. Louis, MO, USA) and disrupting cells by two cycles of alternately in liquid nitrogen and room temperature. Intracellular NADH and NAD^+^ were quantified using the NAD/NADH Quantification Kit according to the manufacturer’s instructions.

## Abbreviations

ADH: alcohol dehydrogenase
ED: Entner–Doudoroff
HimA: integration host factor subunit alpha
NAD: oxidized nicotinamide adenine dinucleotide
NADH: reduced nicotinamide adenine dinucleotide
PBS: phosphate buffer saline
PDC: pyruvate decarboxylase

## References

[1] Panesar PS, Marwaha SS, Kennedy JF (2006). *Zymomonas mobilis*: an alternative ethanol producer. J Chem Technol Biotechnol.

[2] Sahm H, Bringer-Meyer S, Sprenger GA (2006). The genus zymomonas. Prokaryotes.

[3] Seo JS, Chong H, Park HS, Yoon KO, Jung C, Kim JJ, Hong JH, Kim H, Kim JH, Kil JI (2005). The genome sequence of the ethanologenic bacterium *Zymomonas mobilis* ZM4. Nat Biotechnol.

[4] Yang S, Pappas KM, Hauser LJ, Land ML, Chen GL, Hurst GB, Pan C, Kouvelis VN, Typas MA, Pelletier DA (2009). Improved genome annotation for *Zymomonas mobilis*. Nat Biotechnol.

[5] He MX, Wu B, Qin H, Ruan ZY, Tan FR, Wang JL, Shui ZX, Dai LC, Zhu QL, Pan K (2014). *Zymomonas mobilis*: a novel platform for future biorefineries. Biotechnol Biofuels.

[6] Ito T, Nakashimada Y, Senba K, Matsui T, Nishio N (2005). Hydrogen and ethanol production from glycerol-containing wastes discharged after biodiesel manufacturing process. J Biosci Bioeng.

[7] Park SC, Baratti J (1993). Effects of potassium chloride on ethanol production by an osmotolerant mutant of *Zymomonas mobilis*. Appl Microbiol Biotechnol.

[8] Ranatunga TD, Jervis J, Helm RF, McMillan JD, Wooley RJ (2000). The effect of overliming on the toxicity of dilute acid pretreated lignocellulosics the role of inorganics, uronic acids and ether-soluble organics. Enzyme Microb Technol.

[9] Vriesekoop F, Rasmusson M, Pamment NB (2002). Respective effects of sodium and chloride ions on filament formation and growth and ethanol production in *Zymomonas mobilis* fermentations. Lett Appl Microbiol.

[10] Franden MA, Pilath HM, Mohagheghi A, Pienkos PT, Zhang M (2013). Inhibition of growth of *Zymomonas mobilis* by model compounds found in lignocellulosic hydrolysates. Biotechnol Biofuels.

[11] Skerker JM, Leon D, Price MN, Mar JS, Tarjan DR, Wetmore KM, Deutschbauer AM, Baumohl JK, Bauer S, Ibanez AB (2013). Dissecting a complex chemical stress: chemogenomic profiling of plant hydrolysates. Mol Syst Biol.

[12] Zhu L, Li Y, Cai Z (2015). Development of a stress-induced mutagenesis module for autonomous adaptive evolution of *Escherichia coli* to improve its stress tolerance. Biotechnol Biofuels.

[13] Wallace-Salinas V, Gorwa-Grauslund MF (2013). Adaptive evolution of an industrial strain of *Saccharomyces cerevisiae* for combined tolerance to inhibitors and temperature. Biotechnol Biofuels.

[14] Mohagheghi A, Linger J, Smith H, Yang S, Dowe N, Pienkos PT (2014). Improving xylose utilization by recombinant *Zymomonas mobilis* strain 8b through adaptation using 2-deoxyglucose. Biotechnol Biofuels.

[15] Mohagheghi A, Linger JG, Yang S, Smith H, Dowe N, Zhang M, Pienkos PT (2015). Improving a recombinant *Zymomonas mobilis* strain 8b through continuous adaptation on dilute acid pretreated corn stover hydrolysate. Biotechnol Biofuels.

[16] Shui ZX, Qin H, Wu B, Ruan ZY, Wang LS, Tan FR, Wang JL, Tang XY, Dai LC, Hu GQ, He MX (2015). Adaptive laboratory evolution of ethanologenic *Zymomonas mobilis* strain tolerant to furfural and acetic acid inhibitors. Appl Microbiol Biotechnol.

[17] Hayes F (2003). Transposon-based strategies for microbial functional genomics and proteomics. Annu Rev Genet.

[18] Goryshin IY, Reznikoff WS (1998). Tn5 in vitro transposition. J Biol Chem.

[19] Pappas K-M, Galani I, Typas MA (1997). Transposon mutagenesis and strain construction in *Zymomonas mobilis*. J Appl Microbiol.

[20] Deutschbauer A, Price MN, Wetmore KM, Tarjan DR, Xu Z, Shao W, Leon D, Arkin AP, Skerker JM (2014). Towards an informative mutant phenotype for every bacterial gene. J Bacteriol.

[21] Zhang X, Wang T, Zhou W, Jia X, Wang H (2013). Use of a Tn5-based transposon system to create a cost-effective *Zymomonas mobilis* for ethanol production from lignocelluloses. Microb Cell Fact.

[22] Jia X, Wei N, Wang T, Wang H (2013). Use of an EZ-Tn5-based random mutagenesis system to create a *Zymomonas mobilis* with significant tolerance to heat stress and malnutrition. J Ind Microbiol Biotechnol.

[23] Hayashi T, Kato T, Watakabe S, Song W, Aikawa S, Furukawa K (2015). Respiratory chain provides salt stress tolerance by maintaining a low NADH/NAD^+^ ratio in *Zymomonas mobilis*. Microbiology.

[24] Kalnenieks U (2006). Physiology of *Zymomonas mobilis*: some unanswered questions. Adv Microb Physiol.

[25] Rubiano-Labrador C, Bland C, Miotello G, Armengaud J, Baena S (2015). Salt stress induced changes in the exoproteome of the halotolerant bacterium *Tistlia consotensis* deciphered by proteogenomics. PLoS One.

[26] Wood JM (1999). Osmosensing by bacteria: signals and membrane-based sensors. Microbiol Mol Biol Rev.

[27] Empadinhas N, Costa MSd (2008). Osmoadaptation mechanisms in prokaryotes: distribution of compatible solutes. Int Microbiol.

[28] Caimi PG, Chou Y-C, Franden MA, Knoke K, Tao L, Viitanen PV, Zhang M, Zhang Y. *Zymomonas* with improved ethanol production in medium containing concentrated sugars and acetate. pp. Medium: ED2011: Medium: ED.

[29] Caimi Perry G, Tao L, Viitanen Paul V, Chou Y-C, Knoke K, Zhang MIN, Franden Mary ANN, Zhang Y. Process for the production of ethanol from a medium comprising xylose, employing a recombinant zymomonas strain having a reduced himA expression. DU PONT; 2008.

[30] Zhang Q, Piston DW, Goodman RH (2002). Regulation of corepressor function by nuclear NADH. Science.

[31] García-Salcedo JA, Gijón P, Nolan DP, Tebabi P, Pays E (2003). A chromosomal SIR2 homologue with both histone NAD-dependent ADP-ribosyltransferase and deacetylase activities is involved in DNA repair in *Trypanosoma brucei*. EMBO J.

[32] de Souza RF, Aravind L (2012). Identification of novel components of NAD-utilizing metabolic pathways and prediction of their biochemical functions. Mol BioSyst.

[33] Hayashi T, Kato T, Furukawa K (2012). Respiratory chain analysis of *Zymomonas mobilis* mutants producing high levels of ethanol. Appl Environ Microbiol.

[34] Goodman AE, Rogers PL, Skotnicki ML (1982). Minimal medium for isolation of auxotrophic *Zymomonas* mutants. Appl Environ Microbiol.

[35] Tan G, Gao Y, Shi M, Zhang X, He S, Chen Z, An C (2005). Site finding-PCR: a simple and efficient PCR method for chromosome walking. Nucleic Acids Res.

[36] Kuwayama H, Obara S, Morio T, Katoh M, Urushihara H, Tanaka Y (2002). PCR-mediated generation of a gene disruption construct without the use of DNA ligase and plasmid vectors. Nucleic Acids Res.

[37] Yang S, Franden MA, Brown SD, Chou YC, Pienkos PT, Zhang M (2014). Insights into acetate toxicity in *Zymomonas mobilis* 8b using different substrates. Biotechnol Biofuels.

[38] Tan FR, Dai LC, Wu B, Qin H, Shui ZX, Wang JL, Zhu QL, Hu QC, Ruan ZY, He MX (2015). Improving furfural tolerance of *Zymomonas mobilis* by rewiring a sigma factor RpoD protein. Appl Microbiol Biotechnol.

[39] M-X He, Wu B, Shui Z-X, Hu Q-C, Wang W-G, Tan F-R, Tang X-Y, Zhu Q-L, Pan K, Li Q, Su X-H (2012). Transcriptome profiling of *Zymomonas mobilis* under ethanol stress. Biotechnol Biofuels.

[40] Bradford MM (1976). A rapid and sensitive method for the quantitation of microgram quantities of protein utilizing the principle of protein-dye binding. Anal Biochem.

[41] Shin HS, Rogers PL (1995). Biotransformation of benzeldehyde to L-phenylacetylcarbinol, an intermediate in L-ephedrine production, by immobilized *Candida utilis*. Appl Microbiol Biotechnol.

